# Subcutaneous Emphysema Following Periodontal Interventions: A Comprehensive Literature Review and Clinical Considerations

**DOI:** 10.3390/dj14030163

**Published:** 2026-03-11

**Authors:** Ayse Gokce Sahin, Mazlum Bulent Kurtis

**Affiliations:** Department of Periodontology, Faculty of Dentistry, Gazi University, 06490 Ankara, Turkey; mbulent@gazi.edu.tr

**Keywords:** subcutaneous emphysema, air-abrasive devices, periodontal therapy, pneumomediastinum, patient safety

## Abstract

**Background/Objectives**: Subcutaneous emphysema is a rare but potentially life-threatening complication of dental procedures caused by the penetration of pressurized air into submucosal tissues and its spread through cervicofacial and mediastinal spaces. This review aimed to summarize all reported cases of subcutaneous emphysema following periodontal interventions and to identify procedure and device-related etiologic factors associated with its occurrence. **Methods**: A comprehensive literature search was conducted, and case reports published between 1957 and 2025 were included without language restrictions. Cases related to trauma, maxillofacial surgery, endodontic, restorative, or prosthetic procedures were excluded. **Results**: A total of 34 publications reporting 36 clinical casesmet the inclusion criteria. The median patient age was 48 years (range: 8–76), and 66.7% of cases occurred in female patients. Air-powder abrasive devices were the most frequently implicated etiologic factor (66.7%), followed by dental lasers (11.1%) and air-water syringes (8.3%). Mediastinal spread was reported in 41.7% of cases. Most patients received prophylactic antibiotic therapy, and the median resolution time was 5 days (range: 3–14). **Conclusions**: Subcutaneous emphysema following periodontal interventions is most frequently reported in association with the use of pressurized air-driven devices, particularly air-powder abrasive systems. Although the clinical course described in the included cases was generally benign, the observed patterns highlight the relevance of procedural and device-related factors in the development and extent of this complication.

## 1. Introduction

Subcutaneous emphysema is a rare but potentially life-threatening complication of dental procedures and other interventions performed in the head and neck region [1,2]. It develops when pressurized air breaches the disrupted intraoral barrier, penetrating the connective tissue and subsequently spreading into the adjacent fascial spaces. Through these spaces, air may reach the mediastinal or even cranial cavities, potentially leading to infectious complications by carrying microbial agents from the intraoral region [3]. Clinically, subcutaneous emphysema typically presents as a sudden onset of localized swelling accompanied by palpable crepitation and patient discomfort [4]. The differential diagnosis includes angioedema secondary to an allergic reaction, hematoma, and infection, all of which may present with similar clinical findings [3,5].

Advances in dental technology and the increasing variety of dental devices have led to a concerning rise in iatrogenic complications, particularly subcutaneous emphysema during surgical and non-surgical dental procedures [5]. Iatrogenesis refers to complications that occur as unintended outcomes of therapeutic interventions, and periodontal therapy is no exception to this phenomenon [6,7]. While the earliest case of subcutaneous emphysema associated with a dental procedure was described by Turnbull in 1900 during a tooth extraction [8], the first case related to a periodontal procedure was reported by McClendon in 1961, occurring during the examination of a periodontal pocket, with the air-water syringe suggested as the probable cause [9].

In the classification of complications and treatment errors in non-surgical periodontal therapy proposed by Graziani et al. [6], emphysema is categorized as an intraoperative extraoral complication that, although rarely reported following periodontal interventions, may have potentially life-threatening consequences. This complication most commonly arises during dental procedures involving pressurized air devices, whereby air penetrates submucosal spaces through a disrupted epithelial barrier or areas with compromised tissue integrity. In addition, forceful coughing or nose blowing by the patient may also precipitate this outcome even hours after the procedure. Owing to the close anatomical relationship of the head and neck with vital regions, the air introduced in the tissues may travel through various fascial planes particularly from mandibular teeth which directly communicates with submandibular space, subsequently the air may spread to the parapharyngeal and retropharyngeal spaces, eventually reaching the ‘danger space’ and extending toward the mediastinum, a pathway also known to facilitate the translocation of oropharyngeal infections into the thorax [10,11]. Air may also track superiorly into the cranial cavity, potentially leading to the development of pneumocephalus. As described by Bruckmann et al. [12], air introduced during procedures in the maxillary posterior region may spread along the pterygopalatine fossa, pass through the inferior orbital fissure, and reach the intracranial cavity via the pterygoid canal or the foramen rotundum.

The etiology, diagnosis, and management of subcutaneous emphysema associated with dental procedures have been discussed in the reviews by Heyman (1995) [13], McKenzie (2009) [10], and Jones (2021) [5]. Historically, most reported cases were attributed to air-driven devices used during surgical tooth extractions. However, in contemporary periodontal and peri-implant practice, both surgical and non-surgical procedures frequently involve devices that operate with pressurized air, including air-powder abrasive systems, air-driven instruments, air-water syringes, and lasers equipped with an air-cooling mechanism [10,14]. While mechanical disruption and removal of microbial biofilm remain the cornerstone of periodontal and peri-implant therapy, subcutaneous emphysema has emerged as a recognized complication associated with the expanding use of air-powder abrasive devices, particularly in the maintenance care and in the prevention and management of peri-implant diseases [6,15,16,17]. Despite its potentially alarming clinical presentation, subcutaneous emphysema generally follows a benign clinical course, with resolution typically occurring within 3 to 5 days without subsequent morbidity. In most cases, antibiotic therapy is administered to prevent the occurrence of secondary infection [6].

Given that different dental specialties employ devices that operate on diverse mechanical principles, a generalized approach to emphysema prevention may not effectively address the risks associated with this complication. To date, no review has comprehensively focused on subcutaneous emphysema occurring specifically after periodontal interventions.

Therefore, the present review aims to:

- Summarize all reported cases of subcutaneous emphysema following periodontal interventions;
- Identify the possible procedural, operator, device and patient-related etiologic factors; and
- Discuss the clinical implications and preventive considerations of this potentially life-threatening complication based on patterns observed in the reported cases

## 2. Materials and Methods

This manuscript is a comprehensive descriptive literature review of published case reports describing subcutaneous emphysema following surgical or non-surgical periodontal and peri-implant interventions. Given the rarity of this complication and the predominance of single-case publications in the literature, a modified search strategy was employed to retrieve relevant case reports, including those published in low-visibility or non-indexed journals.

Publications were identified through searches of the PubMed database and the Google Scholar search engine, complemented by manual screening of the reference list of relevant articles, non-indexed journals, conference proceedings and other gray literature sources. The inclusion of these additional sources was intended to minimize the risk of missing isolated case reports that may not be captured by conventional bibliographic databases.

The PubMed search strategy incorporated both MeSH terms and free text keywords, as detailed in Appendix A (Table A1). The search terms and their combinations included: “subcutaneous emphysema”, “cervicofacial emphysema”, “periodontal treatment”, “iatrogenic complication”, “pneumomediastinum” and “air-flow”. As a comprehensive review published in 1957 [18] summarized all cases of soft tissue emphysema reported up to that date, the present review focused only on case reports related to periodontal procedures published between 1957 and 2025. No language restrictions were applied, and only articles with full text availability were included to ensure accurate data extraction.

### Study Selection and Data Extraction

Case reports were included if they described an incident of subcutaneous emphysema occurring after a surgical or non-surgical periodontal or peri-implant treatment procedure. Reports of subcutaneous emphysema associated with trauma; maxillofacial surgery; endodontic, prosthetic or restorative procedures; or emphysema arising from other medical causes were excluded. Duplicate records were removed manually.

**Figure 1:**
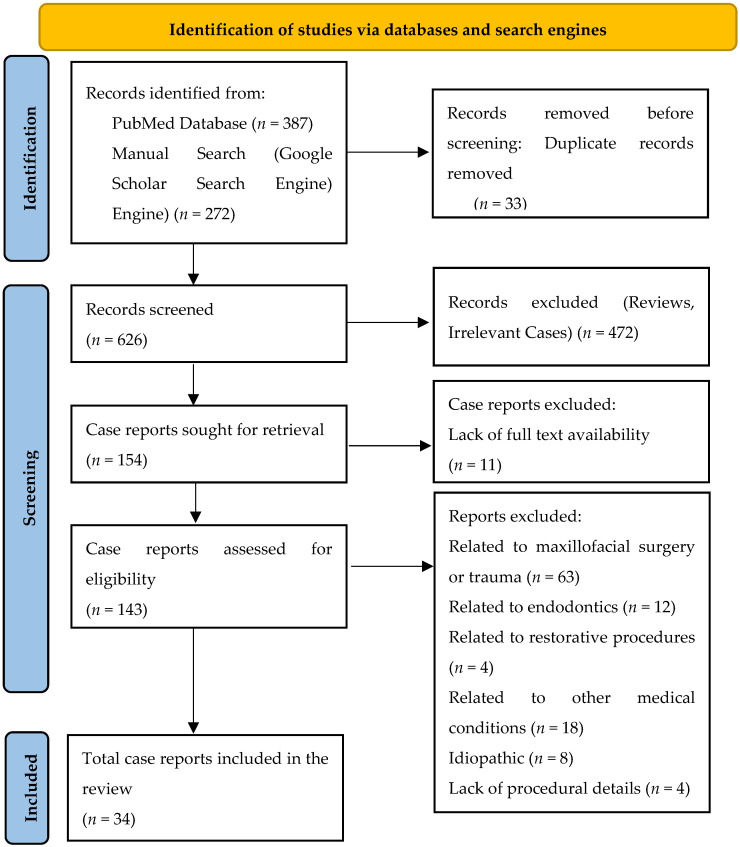
PRISMA 2020 flow diagram illustrating the screening and selection process of the case reports included in the current review [19].

Screening of titles, abstracts, and full texts was conducted to determine eligibility. Case reports published in languages other than English or Turkish were translated using artificial intelligence–assisted translation tools to ensure accurate data extraction. For each included case report, the following data were extracted and summarized in Table 1: patient demographics (age, sex), affected tooth or region, type of periodontal procedure, presumed etiology or device, anatomical distribution of emphysema, treatment, and resolution time. When the case data were incomplete or not clearly stated, missing information was recorded as “insufficient data”. Although this review was not designed as a systematic review, a modified PRISMA 2020 [19] flow diagram (Figure 1) is presented in the Results section to transparently illustrate the source identification and selection process. A formal risk of bias assessment was not applicable due to the descriptive nature of individual case reports.

## 3. Results

### 3.1. Study Selection

Overall, 659 records were identified through electronic database searching (PubMed, *n* = 387) and manual screening of search engines (Google Scholar) (*n* = 272). After the manual removal of 33 duplicate records, 626 studies were screened based on their titles and abstracts, resulting in the exclusion of 472 records. Out of the 154 case reports, 11 were excluded due to the lack of full-text availability. A total of 143 case reports underwent eligibility evaluation, of which 109 were excluded for the following reasons: emphysema related to maxillofacial surgery or trauma (*n* = 63), endodontic origin (*n* = 12), restorative or prosthetic procedures (*n* = 4), emphysema associated with other medical conditions (*n* = 18), idiopathic cases (*n* = 8), and insufficient procedural information (*n* = 4). In total, 34 publications reporting 36 clinical cases fulfilled the eligibility criteria and were included in this review.

Table 1 provides a chronological summary of all included cases outlining the key clinical variables relevant to understanding the mechanisms, presentation, and management of subcutaneous emphysema in periodontal practice. A total of 34 publications reporting 36 clinical cases of subcutaneous emphysema that occurred following periodontal interventions between 1957 and 2025 were identified. Among the 36 reported cases, seven (19.4%) were reported before the year 2000. The demographic characteristics of the cases are summarized in Table 2, showing that 24 patients were female (66.7%) and 12 were male (33.3%), with a median age of 48 (range: 8–76) years.

### 3.2. Procedural Characteristics and Anatomical Distribution

Subcutaneous emphysema was reported during surgical procedures in nine cases (25.0%), and mediastinal distribution of emphysema was documented in 15 cases (41.7%). The origin of the emphysema was identified as the mandible in 17 cases (47.2%) and the maxilla in 17 cases (47.2%), while in two cases (5.6%), the site of origin was not specified. These procedural characteristics and anatomical distribution patterns are summarized in Table 2.

### 3.3. Device-Related Etiology

Regarding the probable etiology or device associated with subcutaneous emphysema, air-powder abrasive devices were the most frequently associated factor, reported in 24 cases (66.7%). Dental lasers were implicated in four cases (11.1%), air-water syringes in three cases (8.3%), air-driven high-speed handpieces in two cases (5.6%), and ultrasonic scalers and non-professional oral irrigators in one case (2.8%). In another case (2.8%), the device was not identified. These device-related etiologic factors are summarized in Table 2.

### 3.4. Antimicrobial Therapy and Clinical Outcomes

The antimicrobial therapy approaches and healing durations reported in the reviewed case reports are presented in Table 3 and Table 4. Out of the 36 reported patients, four (11.1%) did not receive any antibiotic therapy, seven (19.4%) were treated with unidentified antibiotics, and one (2.8%) had no information regarding antibiotic use. The remaining 24 patients (66.7%) received identified antibiotic treatment. Among the 24 patients who received identified antibiotics, 17 (70.8%) were treated with monotherapy, while seven (29.2%) received combined antibiotic regimens. Within the monotherapy group, penicillin was the most commonly prescribed (eight cases, 47.1%), followed by macrolides (three cases, 17.6%), cephalosporins (two cases, 11.8%), lincosamide (two cases, 11.8%), and tetracycline and quinolone (each used in 1 case, 5.9%). Among patients who received combined antibiotic therapies, three (42.9%) were treated with penicillin + nitroimidazole, two (28.6%) with cephalosporin + penicillin, one (14.3%) with cephalosporin + lincosamide and one with cephalosporin + nitroimidazole (14.3%). In addition to antimicrobial therapy, analgesics, steroids, and supplemental oxygen were administered as needed according to the patient’s clinical condition. The overall median resolution time was 5 days (range: 3–14). Three cases were excluded from this analysis due to insufficient data.

## 4. Discussion

### 4.1. Etiology

To contextualize the findings of the present case-based review, the etiologic patterns observed in subcutaneous emphysema following periodontal interventions were compared with those reported in previous reviews addressing dental procedures in general. When examining the reviews on subcutaneous emphysema as a complication of dental procedures chronologically, Heyman et al. [13] reported that between 1960 and 1993, 72% of cases were associated with high-speed devices and occurred during tooth extraction procedures and that 75% of the affected sites were located in the mandible. Jones et al. [5] indicated that, among the cases reviewed between 1993 and 2020, 51.1% were attributed to air-driven handpieces used during tooth extraction, while 3.7% and 3.0% were caused by air polishing devices and lasers, respectively. In contrast to these findings in previous reviews, the current evaluation of subcutaneous emphysema cases following periodontal interventions shows that 24 of the 36 reported clinical cases (66.7%) were associated with air-powder abrasive devices.

### 4.2. Clinical Presentation and Diagnosis

Across the included case reports, subcutaneous emphysema typically presented with the sudden onset of cervicofacial swelling accompanied by palpable crepitation and patient discomfort following periodontal interventions. A careful differential diagnosis is required, particularly to distinguish it from conditions with similar findings such as anaphylaxis, angioedema and infection, as also noted in previous reviews of dental procedure–related emphysema [5,10,13]. In the included case reports, diagnosis was primarily based on clinical examination. In selected cases with suspected mediastinal involvement, additional clinical signs such as chest discomfort or characteristic auscultatory findings (Hamman’s sign) have been described in the literature, and imaging modalities including radiography or computed tomography were used selectively to evaluate the extent of air dissemination and to exclude associated thoracic involvement [3,5,24,44]. In a subset of the included cases, laboratory parameters including C-reactive protein and complete blood count were evaluated to assess the possibility of an underlying infection [28,32,44].

### 4.3. Management

The potential translocation of oral microorganisms through a compromised mucosal barrier into the underlying tissues followed by their spread through communicating fascial spaces has been discussed as a possible risk factor for secondary infection in cases of subcutaneous emphysema. Although no guideline or consensus currently exists regarding the management and antimicrobial treatment of subcutaneous emphysema, several authors have suggested the use of antibiotics to prevent secondary infection, along with NSAIDs for pain control, supplemental oxygen (O_2_), and in some cases, corticosteroids [3,6,44,49,50,51]. Among the reviewed 34 publications reporting 36 clinical cases, clinicians reported prophylactic antibiotic administration in 31 of the patients (86.1%). Out of the 24 patients that received identified antimicrobial therapy, 70.8% received monotherapy and 29.2% received combination therapy, with the most common regimen being penicillin + nitroimidazole. In a previous review [13,52], it was reported that penicillin or ampicillin was administered in 55 of the 75 cases (73%) and that subcutaneous emphysema arising from dental procedures generally follows a benign clinical course. In the reviewed case reports, patients were closely monitored until complete resolution, and hospitalization was reported mainly in severe cases. The median resolution time was 5 days, and available reports indicate that spontaneous recovery typically begins within 3–5 days and is generally completed within 7–10 days [3,10,14,24,53].

### 4.4. Patient Related Factors

The review of 34 publications reporting 36 clinical casea revealed that 24 patients (66.7%) were female, a distribution consistent with the retrospective study conducted by Saito et al. [14]. In their investigation, seven of the 11 reported cases occurred in women, and the authors suggested that this pattern may be related to the higher proportion of subcutaneous fat in females [54]. They further emphasized that the subcutaneous fat-rich nature of the head and neck region may facilitate the spread of emphysema in these areas, whereas the association between age and the occurrence of this complication appears to be weak [14,55]. In addition, behaviors such as extreme nose blowing or coughing have been reported as contributing factors in isolated cases, and notably, incorrect use of a home oral irrigator was identified as a precipitating factor in one of the reviewed reports [20,56].

The cases included in the present review were published between 1957 and 2025, with seven cases (19.4%) reported before the year 2000. This chronological pattern suggests that reports of subcutaneous emphysema have increased over time, potentially reflecting the wider use of air-driven and technology-assisted dental devices.

### 4.5. Device-Related Factors

A review of the published case reports and the corresponding user manuals of air-abrasive devices revealed potential discrepancies between manufacturer recommendations and clinical application, which may contribute to iatrogenic complications. Table 5 summarizes key manufacturer recommendations regarding application angle, distance, duration, as well as the clinical situations in which air-abrasive device use should be avoided or approached with caution. Across different manufacturers, common safety principles include maintaining an operating distance of approximately 2–5 mm, avoiding directing toward the gingival margin, and using continuous sweeping motions. Most manuals caution against device use in the presence of severe inflammation, bleeding, pus discharge, or insufficient keratinized tissue and recommend limiting subgingival application to short durations. Deviation from these recommendations, particularly inappropriate angulation or excessive application time, may facilitate the penetration of pressurized air into deeper tissues and has been reported in association with cases of subcutaneous emphysema [57,58,59,60,61,62].

Concerning dental lasers, devices operating with air-projection systems have also been implicated in the development of subcutaneous emphysema. Although the air pressure generated by lasers such as Er:YAG, CO_2_, and Nd:YAG is generally lower than that of air-driven handpieces, air circulation is often maintained for cooling purposes during soft tissue procedures. In the reported cases, prolonged use or subgingival application of laser systems with active air-cooling systems has been suggested as a potential contributing factor to emphysema development [26,28,31,63].

### 4.6. Operator Related Factors

The occurrence of subcutaneous emphysema appears to be influenced by the interaction between device-related risk factors and operator-dependent practices. An analysis of the included case reports summarized in Table 1 indicates that several operator-related circumstances were repeatedly associated with the development of this complication. In particular, the use of air-abrasive devices after curettage when the epithelial barrier had been disrupted [20,22,32,35,41], or the application of air-driven or air-abrasive devices in the presence of an open mucoperiosteal flap [21,24,42], were frequently reported circumstances. Other contributing conditions included the use of powders with large abrasive particles [3,24,29,34,36,37,38,43,45], insufficient supporting alveolar bone around teeth or implants [23,27], and performing incisions with CO_2_ lasers [26,28,30,31] that work with an air-cooling principle in tissues with reduced resistance. The presence of fragile tissues, lack of attached gingiva [12,25,32,38], performing air-abrasive devices despite the purulent discharge, damage to tissue integrity just before exposure to compressed air [23,35,47], and incorrect angulation of air-abrasive devices designed for supragingival use toward the gingival margin and sulcus were likewise observed [9,20,25,37,46].

Overall, these findings suggest that operator technique, timing of device application, and local tissue conditions, including the integrity of the epithelial barrier, the presence of adequate keratinized tissue, and the mechanical resilience of gingival fibers, represent recurring factors influencing the risk of subcutaneous emphysema following periodontal interventions.

In both supra and subgingival air-abrasive applications, the physicochemical properties of the powder, particularly particle size, have been identified as relevant procedural factors. Low-abrasive powders with smaller particle sizes, such as erythritol [64,65,66,67,68,69], glycine [70,71,72], or trehalose [73], are commonly used for subgingival application [69,74], whereas larger particles have been associated with increased soft tissue damage in experimental studies [75]. The extent of tissue response has been shown to depend not only on particle size but also on the degree of local inflammation [6].

In sites exhibiting moderate to severe inflammation, reduced tissue resistance related to collagen fiber degradation may facilitate the penetration of pressurized air into the periodontal or peri-implant sulcus, thereby increasing the risk of air dissemination into adjacent soft tissue compartments [76]. In the reviewed case reports, this risk was further influenced by local anatomical conditions, including the presence of a long epithelial lining with minimal surrounding structures at peri-implant regions [23,32]. Similarly, in the non-surgical treatment of peri-implantitis using air-polishing devices, the absence of a buccal bone wall and/or a lack of keratinized buccal mucosa has been discussed in several case reports in the present review as a potential risk factor for the development of emphysema [12,23,32,38]. In addition, Bruckmann et al. [12] emphasized that treatment duration may also play a role in the risk of emphysema during subgingival air-polishing procedures. They noted that longer application times could deliver greater amounts of pressurized air toward the base of the defect, potentially reducing tissue resistance.

This review has several limitations that should be considered when interpreting the findings. The available evidence is derived exclusively from published case reports, which limits the ability to draw causal conclusions or to estimate the true incidence of subcutaneous emphysema following periodontal interventions. In addition, variability in case reports, including differences in clinical presentation, device characteristics, and management approaches, limited the possibility of meaningful quantitative analysis.

## 5. Conclusions

This review encompasses cases of subcutaneous emphysema reported in the last 68 years following periodontal interventions. Although this complication remains uncommon, the available evidence indicates that it is predominantly linked to the use of air-driven and air-abrasive devices under specific clinical and procedural conditions. The patterns of case reports identified in this review emphasize the relevance of device characteristics, local tissue conditions, and operator-related factors in the development of subcutaneous emphysema. Increased awareness of these recurring features may support earlier recognition and appropriate clinical management of this complication in periodontal practice.

## Figures and Tables

**Table 1:** Description of subcutaneous emphysema cases followed by periodontal interventions between the years 1960–2025 (listed chronologically) (B: buccal, C: cervical, Cr: cranium, Ct: carotid, CS: cavernous sinus, FT: frontotemporal, IT: infratemporal, M: mediastinum, MS: maxillary sinus, P: parotid space, PA: preauricular, PC: precordium, PM: perimandibular, PO: periorbital, PP: parapharyngeal, PPl: pterygopalatine, RP: retropharynx, PV: prevertebral, SC: subcutaneous, SCl: supraclavicular, SM: submandibular, SO: suboccipital, T: temporal) (Cavi-Jet^®^, Prophy-Jet^®^, Dentsply Sirona, Charlotte, NC, USA) (Air-Flow Master^®^, Air-Flow^®^ Handy 2+, Perioflow^®^, EMS, Nyon, Switzerland) (Kavo Prophyflex^®^, KaVo Dental, Biberach, Germany) (Prophy-Mate^®^, NSK Nakanishi, Kanuma, Tochigi, Japan).

Reference	Year	Age/Gender	Tooth/Region	Procedure	Device	Distribution of Emphysema	Treatment	Resol. (Days)
McClendon et al. [9]	1961	37/M	Mandibular left premolar	Examination of periodontal pocket	Air-water syringe	C, B, PO,	Tetracycline (Insufficient data)	3
Feinstone [20]	1972	47/M	Mandibular right molar	Subgingival scaling and curettage	Water-jet spray device (For home)	PM, B	250 mg penicillin PO 4 times/day for 5 days	3
Snyder et al. [21]	1977	44/M	Mandibular right molar	Open Access Flap Surgery	Air-water syringe	PO, T, PA, B,	Antibiotics (Insufficient data)	4
Finlayson et al. [22]	1988	49/M	Maxillary left molars	Tooth polishing after SRP	Air-powder abrasive device (Cavi-Jet^®^)	PO, PM	Penicillin V Potassium 500 mg PO 4 times/day for 7 days	7
Bergendal et al. [23]	1990	40/F	Mandibular implants	Calculus and debris removal around implants	Air-powder abrasive device (Prophy-Jet^®^)	SC (Insufficient data)	Local application of 0.2% Hibitane^®^, no antibiotics	(Insufficient data)
Van De Velde et al. [24]	1991	55/F	Mandibular implants	Routine peri-implant cleaning	Air-powder abrasive device (Plaque Sweep^®^)	SM	No antibiotics	4
Liebenberg et al. [25]	1997	16/F	Maxillary left molar	Stain and organic debris removal	Air-powder abrasive device (No Data)	PO, PM, M	No antibiotics	7
Frühauf et al. [3]	2004	39/F	Insufficient data	Ultrasonic scaling and stain removal	Air-powder abrasive device (No Data)	PO, SCl, RP, M	Insuffcient data	Insuffcient data
Imai et al. [26]	2009	49/F	Maxillary right first premolar	Mucosal incision for abscess drainage	CO_2_ Laser	B, PO, PP, RP, M	Flomoxef Sodium (IV)	6
Rojas et al. [27]	2009	46/F	Maxillary left first molar	Calculus removal of a tooth that previously had periodontal abscess	Ultrasonic scaler	PO, B, PM	Amoxicillin 500 mg, Metranidazole 500 mg PO 3 times/day 5 days, Penicillin and Metranidazole (IV) NSAID	3
Matsuzawa et al. [28]	2010	64/F	Maxillary right first premolar	Mucosal incision for abscess drainage	Dental laser device	PO, B, P, PP, RP, M	Flomoxef 2 g and Clindamycin 1200 mg/day	6
Strassen et al. [29]	2011	52/M	Maxillary left region	Scaling and polishing	Air-powder abrasive device (No Data)	FT, PO, B, PM, M	Sultamicillin 1.5 g 3 times/day (IV)	4
Suzuki et al. [30]	2012	8/F	Insufficient data	Labial frenectomy	CO_2_ Laser	B, C, RP, SCl, PV, M	Antibiotics (Insufficient data)	7
Mitsunaga et al. [31]	2013	76/F	Maxillary left first molar	Calculus removal and gingival curettage	Er:YAG Laser	B, PM, SM, PP, RP, M, PC	Antibiotics (IV) (Insufficient data)	5
Bassetti et al. [32]	2014	69/M	Maxillary left premolar (implant)	Non-surgical Peri-implantitis treatment (Subgingival curettage, Air-flowing, subgingival irrigation)	Titanium curettes, Air-powder abrasive device (Air-Flow Master^®^) and 3% Hydrogen peroxide solution	T, PO, PM	Amoxicillin/Clavulanic acid 2.2 g IV on the first day, then 1 g 2 times/day PO	7
Lee et al. [33]	2015	59/F	Mandibular left molar	Open Access Flap Surgery for Peri-implantitis	Air-powder abrasive device (Air-Flow^®^)	PO, PM, SM, B, PP, C, M	Amoxicillin/Clavulanic acid Steroid	7
Adamicova et al. [34]	2015	59/F	Maxillary left molars	Scaling and polishing	Air-powder abrasive device (Air-Flow^®^ Handy 2+)	PO, PM, MS SCl, Cr, CS	Clindamycin 20 mg/kg/day 7 days	7
Ozkan et al. [35]	2016	41/F	Mandibular right molar	Gingivectomy around the healing cap of the implant and gingival curettage	Air-water syringe	B, PO, PM	Ciprofloxacin for 7 days and NSAID for 3 days	7
Podzimek et al. [36]	2016	26/F	Mandibular right second molar	Scaling and polishing	Air-powder abrasive device (No Data)	B, PM, C, PV, PP, RP, SO, M	Cefuroxim 1.5 g and Metronidazole 500 mg	4
Toniollo et al. [37]	2016	34/M	Maxillary left molars	Scaling and polishing	Air-powder abrasive device (No Data)	T, PO, PM	Amoxicillin 500 mg and NSAID 3 times/day 7 days	7
Alonso et al. [38]	2017	73/F	Mandibular right quadrant	Routine peri-implant cleaning	Air-powder abrasive device (Kavo Prophyflex^®^)	PO, C, PM	Azithromycin 500 mg/day 3 days PO and Methylprednisolone 40 mg (IM)	4
		43/M	Mandbular mouth floor	Scaling and polishing		C, PM, M	No antibiotics	3–4
		62/F	Mandibular right second molar	Scaling and polishing		C, PO, PM	Azithromycin 500 mg/day 3 days PO and NSAID 600 mg 3 times/day 5 days	5
Bocchialini et al. [39]	2017	65/F	Mandibular left premolar (implant)	Scaling and polishing	Air-powder abrasive device (No Data)	B, PO, C, PM, RP, SCl, M	Antibiotics (IV) (Insufficient data)	4
Paiardi et al. [40]	2018	40/F	Maxillary left molars	Scaling and polishing	Insufficient data	PO, T, PM, PP, C, RP	Azithromycin 500 mg/day PO 8 days	Insufficient data
Lee et al. [23]	2018	51/F	Maxillary right lateral incisor (implant)	Non-surgical Peri-implantitis treatment	Air-powder abrasive device (No Data)	T, IT, Ct, B PO, PM, C, RP, M	Cephalosporin + Piperacillin/Tazobactam (IV) 7 days	10
Acikgoz et al. [41]	2019	31/F	Maxillary right molars	Periodontal debridement	Air-powder abrasive device (Prophy-Mate^®^)	PO, PM, T, RP, PP, C, M	Antibiotics (IV & PO) (Insufficient data)	3
Dodge et al. [42]	2020	67/F	Mandibular anterior region and mouth floor	Alveoloplasty	Air-driven high-speed surgical handpiece	SM (Insufficient data)	Amoxicillin 500 mg 3 times/day 7 days	14
Lau et al. [43]	2020	29/M	Left mandible	Scaling and polishing	Air-powder abrasive device (Prophy-Jet^®^)	P, PM, SM, SCl, C, PV, RP, M	Antibiotics (Insufficient data)	4
La Monaca et al. [24]	2021	65/F	Mandibular left molars (implant)	Open flap debridement for peri-implantitis	Air-powder abrasive device (Kavo Prophyflex^®^)	PO, B, PM	Amoxicillin/Clavulanic acid 1 g 2 times/day and Metronidazole 250 mg 3 times/day 10 days	7–10
Bruckmann et al. [12]	2022	62/F	Maxillary left molars (implant)	Non-surgical Peri-implantitis treatment	Air-powder abrasive device (Perioflow^®^)	PP, PPl, Cr, Ct, CS,	Ceftriaxone 2 g/day and Ampicilline/Sulbactame 3 g/day (IV), Amoxicillin/Clavulanic acid 1 g 2 times/day PO 5 days	3
Shimizu et al. [44]	2022	36/M	Maxillary right molars	Scaling and polishing	Air-powder abrasive device (No Data)	PO, B, PM, C, PP	Cefazolin sodium 1 g/day (IV) 3 days	3
Javorská et al. [45]	2023	33/F	Left maxilla	Scaling and polishing	Air-powder abrasive device (Air-Flow^®^)	PO, T, B, P, C, SCl	Clindamycin 300 mg 3 times/day 7 days	6
Kim et al. [46]	2024	60/F	Maxillary right molars	Scaling and polishing	Air-powder abrasive device (Air-Flow^®^)	PO, T, B, PM, PP, SCl, M	Amoxicillin/Clavulanate 1.2 g and Metronidazole 500 mg (IV)	4
Attia et al. [47]	2024	22/F	Mandibular anterior region	Surgical gingival depigmentation	Air-driven high-speed handpiece	PO, B, T, PM, SM, C	Amoxicillin 500 mg/day PO 7 days	7
Mordi et al. [48]	2025	66/F	Left mandible	Scaling and polishing	Air-powder abrasive device (Perio-Mate^®^)	SM, PM, M, RP, PV	Antibiotics (Insufficient data)	4

**Table 2:** Demographic and clinical characteristics of the subcutaneous emphysema cases following periodontal interventions between the years 1957 and 2025. Percentages were calculated based on the total number of cases (*n* = 36), and due to the heterogeneous age distribution, median values and ranges were used to present the data.

Variable	*n* (%) or Median (Range)
Age (years)	Median (range): 48 (8–76)
Sex	
Female	24 (66.7)
Male	12 (33.3)
Procedure type	
Surgical interventions	9 (25.0)
Anatomical distribution	
Mediastinal spread	15 (41.7)
Site of origin	
Mandible	17 (47.2)
Maxilla	17 (47.2)
Unidentified	2 (5.6)
Device type	
Air-powder abrasive device	24 (66.7)
Dental laser	4 (11.1)
Air-water syringe	3 (8.3)
Air-driven high-speed handpiece	2 (5.6)
Ultrasonic scaler	1 (2.8)
Non-professional oral irrigator	1 (2.8)
Unidentified	1 (2.8)

**Table 3:** Antibiotic administration in the subcutaneous emphysema cases following periodontal interventions between 1957 and 2025.

Antibiotic Administration	*n* (%)
No antibiotic administered	4 (11.1)
Unidentified antibiotic	7 (19.4)
Received identified antibiotic therapy	24 (66.7)
No data available	1 (2.8)

**Table 4:** Antibiotic therapy and clinical outcomes of the subcutaneous emphysema cases.

Antibiotic Therapy (*n* = 24)	*n* (%) or Median (Range)
Monotherapy	17 (70.8)
Combined therapy	7 (29.2)
Monotherapy antibiotic class (*n* = 17)	
Penicillin (β-lactam)	8 (47.1)
Macrolide	3 (17.6)
Cephalosporin (β-lactam)	2 (11.8)
Tetracycline	1 (5.9)
Lincosamide	2 (11.8)
Quinolone	1 (5.9)
Combined antibiotic regimens (*n* = 7)	
Penicillin + Nitroimidazole	3 (42.9)
Cephalosporin + Penicillin	2 (28.6)
Cephalosporin + Lincosamide	1 (14.3)
Cephalosporin + Nitroimidazole	1 (14.3)
Clinical outcomes	
Resolution time (days)	Median (range): 5 (3–14)
No data available	3 (8.3)

**Table 5:** Summary of the manufacturer guidelines for commercially available air-abrasive devices: recommended angulation, distance, duration, and clinical contraindications or precautions.

Device	Angle	Distance	Duration	Avoid or Be Cautious
Air-Flow Master^®^ and Perioflow^®^ (EMS, Nyon, Switzerland)	15–80°	2–5 mm with continuous semi-circular movements	5–10 s per site	Lack of keratinized tissue Pus discharge Intense tissue inflammation Do not use immediately after SRP
Prophy-Mate^®^ and Perio-Mate^®^ (NSK, Nakanishi, Kanuma, Tochigi, Japan)	10–60°	3–5 or 5–10 mm	-	Do not direct toward the periodontal pocket Do not use immediately after SRP Severe inflammation or bleeding
Prophyflex^®^ (KaVo Dental, Biberach, Germany)	60–90°	3–5 mm	-	Must not be directed toward the gingival margin
Cavi-Jet^®^ and Prophy-Jet^®^ (Dentsply Sirona^®^, Charlotte, NC, USA)	60–90°	3–4 mm	-	Must not be applied directly into the periodontal pocket
Lunos^®^ (Dürr Dental, Bietigheim-Bissingen, Germany)	-	Circular motions	5 s per site	Do not aim at the soft tissues or inside the gingival sulcus
PT-B^®^ (Woodpecker, Guilin, China)	30–60°	3–5 mm	5 s per site	-

## Data Availability

This review is based exclusively on data extracted from previously published case reports. No new datasets were generated or analyzed; therefore, data sharing is not applicable.

## Abbreviations

The following abbreviations are used in this manuscript and are listed in alphabetical order.

B	Buccal
C	Cervical
Cr	Cranium
Ct	Carotid
CS	Cavernous Sinus
FT	Frontotemporal
IT	Infratemporal
M	Mediastinum
MS	Maxillary Sinus
P	Parotid Space
PA	Preauricular
PC	Precordium
PM	Perimandibular
PO	Periorbital
PP	Parapharyngeal
PPl	Pterygopalatine
RP	Retropharynx
PV	Prevertebral
SC	Subcutaneous
SCl	Supraclavicular
SM	Submandibular
SO	Suboccipital
T	Temporal

## Appendix A

**Table A1:** Database search strategy and search terms.

Database	Search Strategy
PubMed	“Subcutaneous Emphysema” [Mesh] OR “Subcutaneous emphysema” [Title/Abstract] OR “Cervicofacial emphysema” [Title/Abstract] OR “Iatrogenic emphysema” [Title/Abstract] OR “Dental emphysema” [Title/Abstract] OR Pneumomediastinum [Title/Abstract] AND Dentistry [Mesh] OR “Dental procedures” [Mesh] OR Dental [Title/Abstract] OR Dentistry [Title/Abstract] OR Oral [Title/Abstract] OR “Periodontal diseases” [Mesh] OR “Periodontal therapy” [Title/Abstract] OR “Periodontal procedure” [Title/Abstract] OR “Periodontal intervention” [Title/Abstract]
Manual Search (via Google Scholar)	((“Subcutaneous emphysema” OR “Cervicofacial emphysema” OR “Dental emphysema”) AND (“Periodontal treatment” OR “Periodontal therapy” OR “Air polishing” OR “Air syringe” OR “Air flow” OR “Dental laser”) AND (“Case report” OR “Case series”)) -Trauma
